# Supplementary material for: Development of National Antimicrobial Intravenous-to-Oral Switch Criteria and Decision Aid
Source: J Clin Med. 2023 Mar 7;12(6):2086. doi: 10.3390/jcm12062086 (PMC10058706; doi:10.3390/jcm12062086)
Supplement: Supplementary file 1 [file jcm-12-02086-s001.zip › S2-Pre Delphi_IVOS Criteria.pdf]

Table SX. Forty-two intravenous-to-oral switch (IVOS) criteria from rapid review of the published literature (Literature), informed by hospital IVOS policies (Hospital) and expert advice (Expert) taken forward into the 4-step Delphi process.

IVOS criteria	Source of criteria	Number of Literature papers (n=16) and Trust policies (n=45) (%)	Evidence rating (if Literature) where H=High, M=Medium
**1. Timing of IV antimicrobial review**			
a. Review antimicrobial within **24 hours**	Hospital	11 (24)	
b. Review antimicrobial within **24-48 hours**	Hospital	5 (11)	
c. Review antimicrobial within **48 hours**	Literature<br>Hospital	1 (6)<br>13 (29)	[H]
d. Review antimicrobial within **48-72 hours**	Literature<br>Hospital	3 (19)<br>5 (11)	[M] x2, [H]
**2. Clinical signs and symptoms**			
a. Clinical signs and symptoms should be improving	Literature<br>Hospital	9 (56)<br>29 (64)	[M] x8, [H]
**3. Infection markers**			
a. Temperature should be between 36-38°C	Literature<br>Hospital	2 (13)<br>4 (9)	[M] x2
b. Temperature should be between 36-38 °C past **24 hours**	Literature<br>Hospital	3 (19)<br>14 (31)	[M] x2, [H]
c. Heart rate should be below 90 beats per minute	Literature<br>Hospital	1 (6)<br>13 (29)	[H]
d. Heart rate should be below 90 beats per minute for past **12 hours**	Literature	1 (6)	[M]
e. Heart rate should be below 90 beats per minute for past **24 hours**	Hospital	4 (9)	
f. Blood pressure should be stable	Literature	1 (6)	[M]
g. Blood pressure stable for past **24 hours**	Literature<br>Hospital	2 (13)<br>4 (9)	[M] x2
h. Respiratory rate should be below 20 breaths per minute	Literature<br>Hospital	2 (13)<br>14 (31)	[M], [H]
i. Respiratory rate should be below 20 breaths per minute for past **24 hours**	Literature<br>Hospital	1 (6)<br>6 (13)	[M]
j. White cell count should be normalising	Literature<br>Hospital	3 (19)<br>20 (44)	[M] x2, [H]
k. White cell count should be between 4 and 12 x10^9/L	Literature<br>Hospital	3 (19)<br>6 (13)	[M] x2, [H]
l. White cell count should be between 4 and 12 x10^9/L or normalising	Literature<br>Hospital	1 (6)<br>12 (27)	[M]
m. C-reactive protein should be normalising	Literature<br>Hospital	2 (13)<br>15 (38)	[M] x2
n. C-reactive protein does not reflect severity of illness or the need for IV	Hospital	17 (38)	

antibiotics, and may remain elevated as the infection improves			
**4. Enteral route**			
a. Gastrointestinal tract must be functional	Literature	9 (57)	[M] x8, H
b. Patient can tolerate/swallow oral option	Literature<br>Hospital	9 (57)<br>39 (87)	[M] x6, [H]x3
c. No evidence of malabsorption	Literature<br>Hospital	11 (69)<br>30 (67)	[M] x9, [H] x2
d. No vomiting	Literature<br>Hospital	5 (31)<br>16 (35)	[M] x5
e. There should be a suitable oral option available	Hospital	28 (62)	
f. Check for drug interactions of oral option with patient's other medication	Hospital	4 (9)	
g. Check for allergies to oral option	Hospital	5 (11)	
h. Check patient adherence to oral option	Expert		
**5. Infection exclusions**			
a. Deep-seated infections	Literature	3 (19)	[M] x3
b. Infections requiring high tissue concentration	Literature	3 (19)	[M] x2, [H]
c. Infections requiring prolonged IV therapy	Literature	4 (25)	[M] x3, [H]
d. Critical infection with high risk of mortality	Literature	1 (6)	[M]
e. On microbiology advice	Expert		
f. Endocarditis	Literature<br>Hospital	12 (75)<br>42 (95)	[M] x9, [H] x3
g. Meningitis	Literature<br>Hospital	9 (56)<br>41 (91)	[M] x7, [H] x2
h. Bacteraemia, including *Staph. aureus*	Literature<br>Hospital	9 (56)<br>43 (76)	[M] x9
i. Immunocompromised	Literature<br>Hospital	3 (19)<br>33 (73)	[M] x2, [H]
j. Abscess	Literature<br>Hospital	7 (44)<br>31 (69)	[M] x7
k. Severe or necrotising soft tissue infections	Literature<br>Hospital	5 (31)<br>29 (64)	[M] x5
l. Infections of foreign bodies	Literature<br>Hospital	6 (38)<br>29 (64)	[M] x5, [H]
m. Osteomyelitis	Literature<br>Hospital	6 (38)<br>29 (64)	[M] x5, [H]
n. Septic arthritis	Literature<br>Hospital	5 (31)<br>28 (62)	[M] x5
o. Empyema	Literature<br>Hospital	5 (31)<br>23 (51)	[M] x5
