# Supplementary material for: Development of National Antimicrobial Intravenous-to-Oral Switch Criteria and Decision Aid
Source: J Clin Med. 2023 Mar 7;12(6):2086. doi: 10.3390/jcm12062086 (PMC10058706; doi:10.3390/jcm12062086)
Supplement: Supplementary file 1 [file jcm-12-02086-s001.zip › S3-Delphi_Pilot1st Round_Questionnairev2.pdf]

PILOT/1st ROUND: Questionnaire on Developing an Antimicrobial IV Switch Tool for Adults

Thank you for participating in this questionnaire as part of a consensus gathering (Delphi) process to inform an evidence-based, national antimicrobial intravenous (IV) switch tool for adults. The information you provide will remain anonymous.

The 'Start Smart – Then Focus' antimicrobial stewardship toolkit outlines five antimicrobial prescribing decision options, one of which is to switch antibiotics from intravenous to oral therapy in line with local policies (PHE, 2015). The literature outlines numerous benefits for IV switch to oral; including decreased risk of catheter-related infections, reduced costs and increased patient mobility and comfort (Nguyen *et al.*, 2021). In clinically stable patients, studies report that a timely IV switch is safe and of equal efficacy to the full course of IV therapy (McCarthy and Avent, 2020), with no negative impact on patient outcome (Wongkamhla *et al.*, 2020).

This questionnaire includes antimicrobial IV switch criteria from 45 local Trust or Health Board policies across the UK, the literature and expert opinion. In collaboration with Dr Kieran Hand (National Pharmacy and Prescribing Clinical Lead, NHSEI), we invite you to appraise the criteria in terms of their clinical relevance for safe and effective IV switch and their ease of assessment in the clinical setting of hospitalised adult patients.

The form will take approximately 15 minutes to complete and will be open until **Thursday 10th March 2022**. **(Extended to 15th March 2022)**

Please direct any queries to:

Eleanor Harvey – Chief Pharmaceutical Officer Clinical Fellow, UKHSA (eleanor.harvey@phe.gov.uk)
Dr Diane Ashiru-Oredope – Chair, English Surveillance Programme for Antimicrobial Utilisation and Resistance and UKHSA’s Lead Pharmacist for HCAI, AMR, AMU, Fungal and Sepsis Division (diane.ashiru-oredope@phe.gov.uk)

References

McCarthy and Avent, 2020. Oral or intravenous antibiotics? *Aust Prescr* 2020; 43:45-8
Nguyen *et al.*, 2021. The effect of early switching from intravenous to oral antibiotic therapy: a randomised controlled trial. *J Pharm & Pharmacogn Res* 2021; 9(5):695-703
PHE, 2015. Start Smart – Then Focus: Antimicrobial stewardship toolkit for English hospitals. Available

from: https://www.gov.uk/government/publications/antimicrobial-stewardship-start-smart-then-focus
Wongkamhla *et al.*, 2020. Infectious disease team review using antibiotic switch and discharge criteria shortens the duration of intravenous antibiotic: a single-centre cluster-randomised controlled trial in Thailand. Open Forum Infect Dis. 2020 Nov 4;7(12):ofaa539

Consent

Consent form for professionals in the Delphi process to inform an evidence-based, national antimicrobial IV switch tool for adults. The information you provide will remain anonymous.

1. I consent to participating in this questionnaire and understand that any information I provide will be used to help inform an evidence-based, national antimicrobial IV switch tool for adults *

    ◯ Yes

    ◯ No

About you

Where AM = Antimicrobial; ID = Infectious Diseases

2. Please provide your name *

3. Please provide your email address *

4. What is your profession? *

- ◯ Microbiologist/ID Physician
- ◯ General Physician
- ◯ Surgeon
- ◯ Dentist or Dental Nurse
- ◯ AM/ID Nurse
- ◯ General Nurse/Midwife
- ◯ Specialist AM/ID Pharmacist
- ◯ General Pharmacist
- ◯ Allied Health Professional
- ◯ AM/ID Clinical Researcher
- ◯ General Clinical Researcher
- ◯ Other

5. How many years have you been practising within your profession? *

6. If an AM/ID specialist, for how long have you been in your specialist role? *

- 0-1 year
- 1-2 years
- 2-5 years
- 5-10 years
- 10+ years
- Not applicable

7. Which of the following describes your main area(s) of work? *

- [ ] NHS Acute Trust
- [ ] Health Board
- [ ] NHSEI
- [ ] UKHSA
- [ ] Other Arms Length Body/Organisation
- [ ] Mental Health
- [ ] Community
- [ ] Academic
- [ ] Government
- [ ] General Practice/CCG
- [ ] Clinical Fellow (national/regional)
- [ ] Other

8. If you are a Clinical Fellow, please state your role and organisation prior to the fellowship (e.g. clinical pharmacist at an acute hospital)

9. Which country do you work in? *

- ◯ England
- ◯ Northern Ireland
- ◯ Scotland
- ◯ Wales
- ◯ Other

10. Which region do you work in? *

- ◯ East of England
- ◯ London
- ◯ Midlands
- ◯ North East and Yorkshire
- ◯ North West
- ◯ South East
- ◯ South West
- ◯ National
- ◯ Not applicable

11. What is your gender? *

- ◯ Male
- ◯ Female
- ◯ Prefer not to say

12. PILOT/1st ROUND: Please provide comments on the content of this page, including the question number

Antimicrobial IV switch criteria

Respondents are asked to appraise each criterion via a 5-point Likert scale, with additional options of 'Not applicable' or 'I don't know'.

The questionnaire presents IV switch criteria in 5 sections:

- Timing of IV antimicrobial review
- Clinical signs and symptoms
- Infection markers
- Enteral route
- Infection exceptions

Each section is presented twice:
1) Please consider the clinical **relevance** of criteria for safe and effective IV switch. For each criterion: is it clinically significant and does it need to be met for a safe and effective IV switch? does it apply to hospitalised adults on IV antimicrobials?
2) Please consider the **ease** with which criteria can be assessed in the clinical setting. For each criterion: how achievable is it in practice?

[illegible]

13. For clinically stable patients, in your experience what impact has antimicrobial IV switch had on patient outcomes? *

- ○ Positive
- ○ Negative
- ○ Neither positive nor negative
- ○ I don't know

14. Timing of IV antimicrobial review: How clinically relevant is it to review IV antimicrobials within each specified timeframe? *

	Highly relevant	Relevant	More or less relevant	Not relevant	Not at all relevant	Not applicabl
a. Review antimicrobial within **24 hours**	○	○	○	○	○	○
b. Review antimicrobial within **24-48 hours**	○	○	○	○	○	○
c. Review antimicrobial within **48 hours**	○	○	○	○	○	○
d. Review antimicrobial within **48-72 hours**	○	○	○	○	○	○

15. Timing of IV antimicrobial review: How easy is it for the clinical team to review IV antimicrobials within each specified timeframe? *

	Very easy	Easy	More or less easy	Not easy	Not at all easy	Not applical
a. Review antimicrobial within **24 hours**	◯	◯	◯	◯	◯	◯
b. Review antimicrobial within **24-48 hours**	◯	◯	◯	◯	◯	◯
c. Review antimicrobial within **48 hours**	◯	◯	◯	◯	◯	◯
d. Review antimicrobial within **48-72 hours**	◯	◯	◯	◯	◯	◯

16. Clinical signs and symptoms: How clinically relevant is this criterion for safe and effective IV switch? *

	Highly relevant	Relevant	More or less relevant	Not relevant	Not at all relevant	Not applical
a. Clinical signs and symptoms should be improving	◯	◯	◯	◯	◯	◯

17. Clinical signs and symptoms: How easy is it for the clinical team to assess this criterion for IV switch? *

	Very easy	Easy	More or less easy	Not easy	Not at all easy	Not applical
a. Clinical signs and symptoms should be improving	◯	◯	◯	◯	◯	◯

18. Infection markers: How clinically relevant is each criterion for safe and effective IV switch? *

	Highly relevant	Relevant	More or less relevant	Not relevant	Not at all relevant	Not applical
a. Temperature should be between 36-38°C	◯	◯	◯	◯	◯	◯
b. Temperature should be between 36-38°C for past **24 hours**	◯	◯	◯	◯	◯	◯
c. Heart rate should be below 90 beats per minute	◯	◯	◯	◯	◯	◯
d. Heart rate should be below 90 beats per minute for past **12 hours**	◯	◯	◯	◯	◯	◯
	Highly relevant	Relevant	More or less relevant	Not relevant	Not at all relevant	Not applical

e. Heart rate should be below 90 beats per minute for past **24 hours**	○	○	○	○	○	○
f. Blood pressure should be stable	○	○	○	○	○	○
g. Blood pressure should be stable for past **24 hours**	○	○	○	○	○	○
h. Respiratory rate should be below 20 breaths per minute	○	○	○	○	○	○
i. Respiratory rate should be below 20 breaths per minute for past **24 hours**	○	○	○	○	○	○
j. White cell count should be normalising	○	○	○	○	○	○
k. White cell count should be between 4 and 12 x10^9/L	○	○	○	○	○	○
l. White cell count should be between 4 and 12 x10^9/L or normalising	○	○	○	○	○	○
	Highly relevant	Relevant	More or less relevant	Not relevant	Not at all relevant	Not applical
m. C-reactive						

protein should be normalising	○	○	○	○	○	○
n. C-reactive protein does not reflect severity of illness or the need for IV antibiotics, and may remain elevated as the infection improves	○	○	○	○	○	○

19. Infection markers: How easy is it for the clinical team to assess each criterion for IV switch? *

	Very easy	Easy	More or less easy	Not easy	Not at all easy	Not applical
a. Temperature should be between 36-38°C	○	○	○	○	○	○
b. Temperature should be between 36-38°C for past **24 hours**	○	○	○	○	○	○
c. Heart rate should be below 90 beats per minute	○	○	○	○	○	○
d. Heart rate should be below 90 beats per minute for past **12 hours**	○	○	○	○	○	○
	Very easy	Easy	More or less easy	Not easy	Not at all easy	Not applical

e. Heart rate should be below 90 beats per minute for past **24 hours**	○	○	○	○	○	○
f. Blood pressure should be stable	○	○	○	○	○	○
g. Blood pressure should be stable for past **24 hours**	○	○	○	○	○	○
h. Respiratory rate should be below 20 breaths per minute	○	○	○	○	○	○
i. Respiratory rate should be below 20 breaths per minute for past **24 hours**	○	○	○	○	○	○
j. White cell count should be normalising	○	○	○	○	○	○
k. White cell count should be between 4 and 12 x10^9/L	○	○	○	○	○	○
l. White cell count should be between 4 and 12 x10^9/L or normalising	○	○	○	○	○	○
	Very easy	Easy	More or less easy	Not easy	Not at all easy	Not applical
m. C-reactive						

protein should be normalising	○	○	○	○	○	○
n. C-reactive protein does not reflect severity of illness or the need for IV antibiotics, and may remain elevated as the infection improves	○	○	○	○	○	○

20. Enteral route: How clinically relevant is each criterion for safe and effective IV switch? *

	Highly relevant	Relevant	More or less relevant	Not relevant	Not at all relevant	Not applical
a. Gastrointestinal tract must be functional	○	○	○	○	○	○
b. Patient can tolerate/ swallow oral option	○	○	○	○	○	○
c. No evidence of malabsorption	○	○	○	○	○	○
d. No vomiting	○	○	○	○	○	○
e. There should be a suitable oral option available	○	○	○	○	○	○
f. Check for drug interactions of oral option with patient's other medication	○	○	○	○	○	○
g. Check for allergies to oral option	○	○	○	○	○	○
h. Check patient adherence to oral option	○	○	○	○	○	○

21. Enteral route: How easy is it for the clinical team to assess each criterion for IV switch? *

	Very easy	Easy	More or less easy	Not easy	Not at all easy	Not applical
a. Gastrointestinal tract must be functional	○	○	○	○	○	○
b. Patient can tolerate/ swallow oral option	○	○	○	○	○	○
c. No evidence of malabsorption	○	○	○	○	○	○
d. No vomiting	○	○	○	○	○	○
e. There should be a suitable oral option available	○	○	○	○	○	○
f. Check for drug interactions of oral option with patient's other medication	○	○	○	○	○	○
g. Check for allergies to oral option	○	○	○	○	○	○
h. Check patient adherence to oral option	○	○	○	○	○	○

22. Infection exceptions: How clinically relevant is each criterion to warrant exclusion from using this IV switch tool? (and for referral to clinician in charge of patient's care) *

	Highly relevant	Relevant	More or less relevant	Not relevant	Not at all relevant	Not applical
a. Deep-seated infections	○	○	○	○	○	○
b. Infections requiring high tissue concentration	○	○	○	○	○	○
c. Infections requiring prolonged IV therapy	○	○	○	○	○	○
d. Critical infection with high risk of mortality	○	○	○	○	○	○
e. On microbiology advice	○	○	○	○	○	○
f. Endocarditis	○	○	○	○	○	○
g. Meningitis	○	○	○	○	○	○
h. Bacteraemia, including *Staph. aureus*	○	○	○	○	○	○
i. Immunocompromised	○	○	○	○	○	○
j. Abscess	○	○	○	○	○	○
k. Severe or necrotising soft tissue	○	○	○	○	○	○

Highly relevant Relevant More or less relevant Not relevant Not at all relevant Not applical

infections						
l. Infections of foreign bodies						
m. Osteomyelitis						
n. Septic arthritis						
o. Empyema						

23. Infection exceptions: How easy is it for the clinical team to assess each criterion? *

	Very easy	Easy	More or less easy	Not easy	Not at all easy	Not applical
a. Deep-seated infections						
b. Infections requiring high tissue concentration						
c. Infections requiring prolonged IV therapy						
d. Critical infection with high risk of mortality						
e. On microbiology advice						
f. Endocarditis						
g. Meningitis						

Very easy Easy More or less easy Not easy Not at all easy Not applical

h. Bacteraemia, including *Staph. aureus*	○	○	○	○	○	○
i. Immunocompromised	○	○	○	○	○	○
j. Abscess	○	○	○	○	○	○
k. Severe or necrotising soft tissue infections	○	○	○	○	○	○
l. Infections of foreign bodies	○	○	○	○	○	○
m. Osteomyelitis	○	○	○	○	○	○
n. Septic arthritis	○	○	○	○	○	○
o. Empyema	○	○	○	○	○	○

24. Please use the box below if you would like to suggest any criteria word amendments, please specify which criteria you are referring to (e.g. Question 14a)

25. Please use the box below if you would like to suggest any additional criteria that would add benefit to the national IV switch tool

26. For wider dissemination of questionnaire, which Likert scale wording is most appropriate to be able to determine whether criteria should be met for a safe and effective IV switch?

- ◯ Clinically **relevant**
- ◯ Clinically **significant**
- ◯ Other

27. In your opinion, how feasible is it for the **nursing** team to prompt an IV antimicrobial review in relation to each of the 5 outlined sections? *

	Very feasible	Feasible	More or less feasible	Not feasible	Not at all feasible	Not applical
Timing of IV antimicrobial review	◯	◯	◯	◯	◯	◯
Clinical signs and symptoms	◯	◯	◯	◯	◯	◯
Infection markers	◯	◯	◯	◯	◯	◯
Enteral route	◯	◯	◯	◯	◯	◯
Infection exceptions	◯	◯	◯	◯	◯	◯

28. In your opinion, how feasible is it for the **pharmacy** team to prompt an IV antimicrobial review in relation to each of the 5 outlined sections? *

	Very feasible	Feasible	More or less feasible	Not feasible	Not at all feasible	Not applical
Timing of IV antimicrobial review	○	○	○	○	○	○
Clinical signs and symptoms	○	○	○	○	○	○
Infection markers	○	○	○	○	○	○
Enteral route	○	○	○	○	○	○
Infection exceptions	○	○	○	○	○	○

29. PILOT/1st ROUND: Please provide comments on the content of this page, including the question number

Electronic Prescribing and Medicines Administration (ePMA)

30. In your clinical area of work, do you use ePMA systems? *

- ◯ Yes
- ◯ No
- ◯ Not applicable

31. Has ePMA facilitated, hindered or not affected antimicrobial IV switch? *

- ◯ Facilitated
- ◯ Hindered
- ◯ Not affected
- ◯ I don't know

32. PILOT/1ST ROUND: Please provide comments on the content of this page, including the question number

Further comments

33. Thank you for your time in completing this survey. Please use the box below for any further comments

34. Can we contact you further regarding this project? *

◯ Yes

◯ No

This content is neither created nor endorsed by Microsoft. The data you submit will be sent to the form owner.

Microsoft Forms
