# Supplementary material for: Development of National Antimicrobial Intravenous-to-Oral Switch Criteria and Decision Aid
Source: J Clin Med. 2023 Mar 7;12(6):2086. doi: 10.3390/jcm12062086 (PMC10058706; doi:10.3390/jcm12062086)
Supplement: Supplementary file 1 [file jcm-12-02086-s001.zip › S6-Delphi_All Steps_Criteria Outcomes .pdf]

Table SX. Criteria outcomes of each step in the Delphi process: Step One) Pilot/1st round questionnaire, Step Two) Virtual meeting, Step Three) 2nd round questionnaire and Step 4) Workshop.

**IVOS criteria**	**Step One) Pilot/1st round questionnaire**					**Step Two) Virtual meeting**	**Step Three) 2nd round questionnaire**			**Step Four) Workshop**
	'Relevance' median	Percentage agreement (relevant and very relevant) (%)	'Ease' median	Percentage agreement (easy and very easy) (%)	Outcome	Outcome	Median	Percentage agreement (agree and strongly agree) (%)	Outcome	Outcome
**1. Timing of IV antimicrobial review**										
a. Review antimicrobial within **24 hours**	4	70.83	4	54.17	Accepted	Rephrased into 1e				
b. Review antimicrobial within **24-48 hours**	4.5	91.67	4	66.67	Accepted	Rephrased into 1g				
c. Review antimicrobial within **48 hours**	5	91.67	4	66.67	Accepted	Rephrased into 1h				
d. Review antimicrobial within **48-72 hours**	5	87.50	4.5	75.00	Accepted	Rephrased into 1i				
e. IVOS should be considered **any time after** the first dose of IV antimicrobial is administered						New criteria proposed	4	65.29	Rejected	
f. IVOS should be considered **within 24 hours** of the first dose of IV antimicrobial being administered						Result from rephrase 1a	4	61.98	Rejected	

g. IVOS should be considered **24 hours after** the first dose of IV antimicrobial						Result from rephrase 1b	4	69.83	Rejected	
h. IVOS should be considered **within 48 hours** of the first dose of IV antimicrobial being administered						Result from rephrase 1c	4	78.10	Accepted	Accepted
i. IVOS should be considered **48 hours after** the first dose of IV antimicrobial is administered						Result from rephrase 1d	4	71.49	Rejected	
j. If no IVOS within first 48 hours, review daily thereafter						New criteria proposed	-	88.02	Accepted	Accepted
j. If no IVOS within first 48 hours, review every two days thereafter						New criteria proposed	-	6.20	Rejected	
**2. Clinical signs and symptoms**										
a. Clinical signs and symptoms should be improving	5	91.67	4	58.33	Accepted	Rephrased into 2b				
b. Clinical signs and symptoms are improving						Result from rephrase 2a	5	95.04	Accepted	Rephrased into 2d
c. Clinical signs and symptoms are not worsening						New criteria proposed	3	36.78	Rejected	
d. Clinical signs and symptoms of infection are improving										Result from rephrase 3b
**3. Infection markers**										

a. Temperature should be between 36-38°C	4	54.17	4.5	75.00	Uncertain	Rephrased into 3o				
b. Temperature should be between 36-38°C for past **24 hours**	4	70.83	4.5	70.83	Accepted	Rephrased into 3o				
c. Heart rate should be below 90 beats per minute	3	45.83	4.5	70.83	Rejected	Rephrased into 3q-r				
d. Heart rate should be below 90 beats per minute for past **12 hours**	3	45.83	4	62.50	Rejected	Rejected				
e. Heart rate should be below 90 beats per minute for past **24 hours**	3	41.67	4.5	66.67	Rejected	Rejected				
f. Blood pressure should be stable	4	58.33	4	70.83	Uncertain	Rephrased into 3q-r				
g. Blood pressure should be stable for past **24 hours**	4	58.33	4	66.67	Uncertain	Rejected				
h. Respiratory rate should be below 20 breaths per minute	3.4	50.00	4	66.67	Rejected	Rephrased into 3q-r				
i. Respiratory rate should be below 20 breaths per minute for past **24 hours**	4	54.17	4	62.50	Uncertain	Rejected				
j. White cell count should be normalising	4	54.17	4	66.67	Uncertain	Rephrased into s-t				
k. White cell count should be between 4 and 12 x10^9/L	3	29.17	4	62.50	Rejected	Rejected				
l. White cell count should be between 4 and 12 x10^9/L or normalising	3	45.83	4	62.50	Rejected	Rejected				

m. C-reactive protein should be normalising	3	29.17	4	66.67	Rejected	Rephrased into 3u-v				
n. C-reactive protein does not reflect severity of illness or the need for IV antibiotics, and may remain elevated as the infection improves	3	45.83	3.5	50.00	Rejected	Rejected				
o. Temperature is between 36-38°C						Result from rephrase 3a	3	47.52	Rejected	
p. Temperature is between 36-38°C for the past **24 hours**						Result from rephrase 3b	4	75.21	Accepted	Accepted
q. Early Warning Score (e.g. MEWS, NEWS2) is **improving**						Result from rephrase 3c-i	4	87.19	Accepted	Rephrased into 3w
r. Early Warning Score (e.g. MEWS, NEWS2) is **not worsening**						Result from rephrase 3c-i	3	35.54	Rejected	
s. White Cell Count is **improving**						Result from rephrase 3j	4	76.03	Accepted	Rephrased into 3x
t. White Cell Count is **not worsening**						Result from rephrase 3j	3	34.30	Rejected	
u. C-Reactive Protein is **improving**						Result from rephrase 3m	4	73.55	Accepted	Rephrased into 3y

v. C-Reactive Protein is **not worsening**						Result from rephrase 3m	3	33.47	Rejected	
w. Early Warning Score is **decreasing**										Result from rephrase 3q
x. White Cell Count is **trending towards the normal range**										Result from rephrase 3s
y. C-Reactive Protein is **decreasing**										Result from rephrase 3u
**4. Enteral route**										
a. Gastrointestinal tract must be functional	5	87.50	3	29.17	Accepted	Accepted	5	96.69	Accepted	Rephrased into 4n
b. Patient can tolerate/ swallow oral option	5	87.50	4	62.50	Accepted	Accepted	5	96.69	Accepted	Rephrased into 4o
c. No evidence of malabsorption	4	91.67	3	29.17	Accepted	Accepted	5	95.04	Accepted	Rephrased into 4n
d. No vomiting	4.5	91.67	4	75.00	Accepted	Rephrased into 4i				
e. There should be a suitable oral option available	5	91.67	4	54.17	Accepted	Rephrased into 4j				
f. Check for drug interactions of oral option with patient's other medication	5	91.67	4	70.83	Accepted	Rephrased into 4k				
g. Check for allergies to oral option	5	91.67	4	70.83	Accepted	Rephrased into 4l				
h. Check patient adherence to oral option	4.5	91.67	3	25.00	Accepted	Rephrased into 4m				
i. No vomiting within the last 24 hours						Result from rephrase 4d	4	79.75	Accepted	Accepted

j. Suitable oral switch option available						Result from rephrase 4e	5	98.35	Accepted	Rephrased into 4p
k. No contraindication or clinically significant drug interaction affecting oral switch option						Result from rephrase 4f	5	95.45	Accepted	Rephrased into 4p
l. No clinically significant allergy to oral switch option						Result from rephrase 4g	5	96.69	Accepted	Rephrased into 4p
m. No significant concerns over patient adherence to oral switch option						Result from rephrase 4h	4	91.74	Accepted	Rephrased into 4q
n. Gastrointestinal tract is functioning with no evidence of malabsorption										Result from rephrase 4a and 4c
o. Safe swallow or enteral tube administration										Result from rephrase 4b
p. Suitable oral switch option available, considering oral bioavailability, any clinically significant drug interactions or patient allergies										Result from rephrase 4j-m
q. No significant concerns over patient adherence to oral treatment										Result from rephrase 4m
**5. Infection exceptions (at Step One) > Infection exclusions (at Step Three) > Special consideration (at Step Four)**										

a. Deep-seated infections	4	58.33	3	41.67	Uncertain	Rephrased into 5p				
b. Infections requiring high tissue concentration	4	62.50	2	25.00	Accepted	Rephrased into 5q				
c. Infections requiring prolonged IV therapy	4	70.83	3	33.33	Accepted	Rephrased into 5r				
d. Critical infection with high risk of mortality	4	75.00	3	37.50	Accepted	Accepted	5	92.15	Accepted	Accepted
e. On microbiology advice	4	66.67	4	62.50	Accepted	Rejected				
f. Endocarditis	4	75.00	3	45.83	Accepted	Accepted	5	87.19	Accepted	Accepted
g. Meningitis	4.5	83.33	3	45.83	Accepted	Accepted	5	89.26	Accepted	Accepted
h. Bacteraemia, including *Staph. aureus*	4	75.00	4	54.17	Accepted	Accepted	4.5	86.36	Accepted	Rephrased into 5t
i. Immunocompromised	3.5	50.00	4	62.50	Rejected	Rejected				
j. Abscess	3	41.67	3	45.83	Rephrased to 'Undrained abscess'	Rephrased into 5s				
k. Severe or necrotising soft tissue infections	4	70.83	3	41.67	Accepted	Accepted	5	90.08	Accepted	Accepted
l. Infections of foreign bodies	3.5	50.00	3	33.33	Rejected	Rejected				
m. Osteomyelitis	4	54.17	2	33.33	Uncertain	Accepted	4	77.27	Accepted	Accepted
n. Septic arthritis	4	58.33	3	33.33	Uncertain	Accepted	4	79.34	Accepted	Accepted
o. Empyema	4	54.17	3	37.50	Uncertain	Accepted	4	71.07	Accepted	Accepted
p. Deep-seated infection						Result from	4	81.40	Accepted	Accepted

						rephrase 5a				
q. Infection requiring high tissue concentration						Result from rephrase 5b	4	78.93	Accepted	Accepted
r. Infection requiring prolonged IV therapy						Result from rephrase 5c	5	89.26	Accepted	Accepted
s. Undrained abscess						Result from rephrase 5j	4	69.83	Accepted	Accepted
t. Bloodstream infection										Result from rephrase 5h
